# The Importance of ^1^H-Nuclear Magnetic Resonance Spectroscopy for Reference Standard Validation in Analytical Sciences

**DOI:** 10.1371/journal.pone.0042061

**Published:** 2012-07-27

**Authors:** Dovi Kelman, Anthony D. Wright

**Affiliations:** Department of Pharmaceutical Sciences, College of Pharmacy, University of Hawaii at Hilo, Hilo, Hawaii, United States of America; Università di Napoli Federico II, Italy

## Abstract

This paper highlights the importance of recording at least a ^1^H nuclear magnetic resonance (NMR) spectrum to verify identity of standards used in analyses of organic materials irrespective of source. We show the importance of this approach with an example of a quantitative high-performance liquid chromatography (HPLC) study undertaken with green tea extracts that required the use of several polyphenols as standards. In the course of the study one of these standards [(-)-epigallocatechin, EGC], although having the physical appearance and appropriate HPLC chromatographic behavior of EGC, proved by ^1^H-NMR to be a completely different class of molecule. For us, this raised significant questions concerning validity of many published pieces of research that used quantitative HPLC methods without first performing rigorous validation of the employed standards prior to their use. This paper clearly illustrates the importance of validation of all standards used in analysis of organic materials by recording at least a ^1^H-NMR spectrum of them prior to their use.

## Introduction

Qualitative and quantitative chromatographic analyses are used extensively in all areas of analytical sciences. Due to the high sensitivities of the detection instruments available today the techniques are invaluable in the analysis of environmental samples (soil and water contamination and atmospheric pollution), in chemical ecology studies, for forensic purposes (such as testing for drug residues in blood and urine, for flammable materials in arson samples and traces of poisons or toxic materials), in pharmaceutical and clinical studies, in chemical biology and in virtually any situation where they might find an application [1]. Such techniques are extensively used by the pharmaceutical industry in drug discovery, pre-formulation, pharmacokinetics, drug metabolism, process development, formulation development, technical transfer and manufacturing, for both research and quality control purposes [2]. There are several detection methods commonly used that are coupled to high performance liquid chromatography (HPLC). The most widespread technique used in many laboratories is HPLC coupled with UV/Vis photodiode array detection (HPLC-UV/Vis-PDA) [3]. HPLC coupled to mass spectrometry (LC-MS) has also become prevalent. More recently, HPLC coupled with nuclear magnetic resonance (LC-NMR) has been given more attention, due to progress in pulse field gradient solvent suppression, improvement in probe-head technology, the construction of cryo-probes and high field magnets [3].

**Figure 1:**
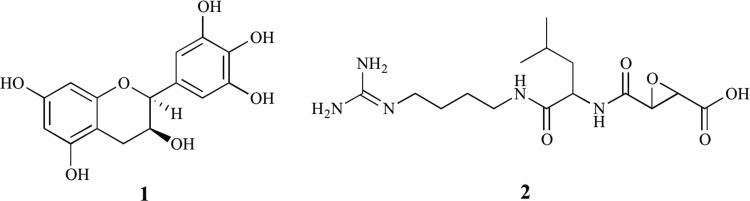
Structural formula of (–)-epigallocatechin (EGC, 1) and -epoxysuccinyl-*trans* *L*-leucylamido-(4-guanidino)-butane (E-64, 2).

One of the key features of quantitative HPLC methods is their use of reference standards, which can be internal or external [1]. Recently, we performed a quantitative HPLC study that looked at various extracts of green tea made from leaves of varying ages and exposed to varying levels of shade for their relative concentrations of six naturally occurring compounds commonly found in them, namely; *L*-theanine, caffeine, and four catechin-polyphenols; (–)-epigallocatechin gallate (EGCG), (–)-epigallocatechin (EGC) (Fig. 1, Structure **1**), (–)-epicatechin (EC), and (–)-epicatechin gallate (ECG). To confirm the identity of compounds within our chromatograms it was necessary to use validated standards as references, in this case ones that were commercially available. In the course of the investigation it was found that one of these standards (EGC, **1**), although having the physical appearance and appropriate HPLC chromatographic behavior of EGC (i.e., HPLC retention time), proved by ^1^H-NMR to be a completely different class of molecule.

For us, this raised significant questions concerning validity of many published pieces of research that used quantitative HPLC methods without first performing rigorous validation of the employed standards prior to their use. Here, we show chemical analyses of the standard EGC as an example that illustrates the importance of validation of all standards used in the analysis of organic materials by recording at least a ^1^H-NMR spectrum of them prior to their use.

## Materials and Methods

### Materials and Sample Preparation

Reference standards of *L*-theanine (>99%, Product No. T6576), caffeine (99%, Product No. C53), EGC (≥95%, Product No. E3768), EC (≥98%, Product No. E4018), and ECG (≥98%, Product No. E3893) were from Sigma-Aldrich Corp. (St. Louis, MO, USA). EGCG (98%, Product No. 02566-34) and another reference standard of EGC (98%, Product No. 02564-54) were from Nacalai USA (San Diego, CA, USA).

Green tea extracts were prepared from fresh *Camellia sinensis* leaves collected from “Mauna Kea Tea”, a tea plantation on the Big Island of Hawaii. Each sample was micro-waved three times for 30 seconds each to quench all enzyme activity in collected leaves. The samples were then dried for 15 minutes at 85°C in a laboratory oven. Dried samples, after leaf stems had been removed, were powdered and weighed. Resultant powders were extracted at ambient temperature (25°C) in a mixture of methanol and water (70∶30 v/v) for 30 min and then filtered prior to HPLC analysis.

**Figure 2:**
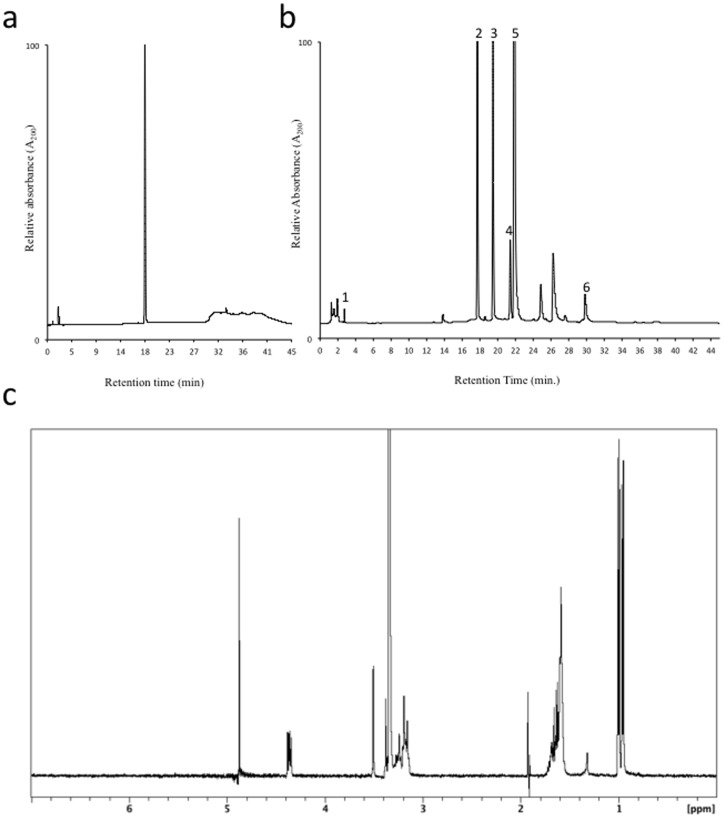
EGC reference standard from Sigma-Aldrich Corp. (Product No. E3768) and tea extract. (**a**) HPLC chromatogram of EGC reference standard from Sigma-Aldrich Corp.; (**b**) HPLC chromatogram of green tea extract. Peak identification: (1) L-theanine; (2) (–)-epigallocatechin (EGC); (3) caffeine; (4) (–)-epicatechin (EC); (5) (–)-epigallocatechin gallate (EGCG); (6) (–)-epicatechin gallate (ECG); and (**c**) ^1^H-NMR spectrum, record in CD_3_OD at 400 MHz, and with peak suppression of the HOD peak at approximately 4.87 ppm, at 25°C, of supposed EGC (ca. 5 mg/mL) from Sigma-Aldrich Corp. (Product No. E3768).

Ultra-pure water for HPLC was prepared using a Millipore (Billerica, MA, USA) Direct-Q purification system. HPLC-grade acetonitrile and methanol were purchased from Fisher Scientific (Pittsburgh, PA, USA). CD_3_OD was purchased from Cambridge Isotope Laboratories (Andover, MA, USA).

**Figure 3:**
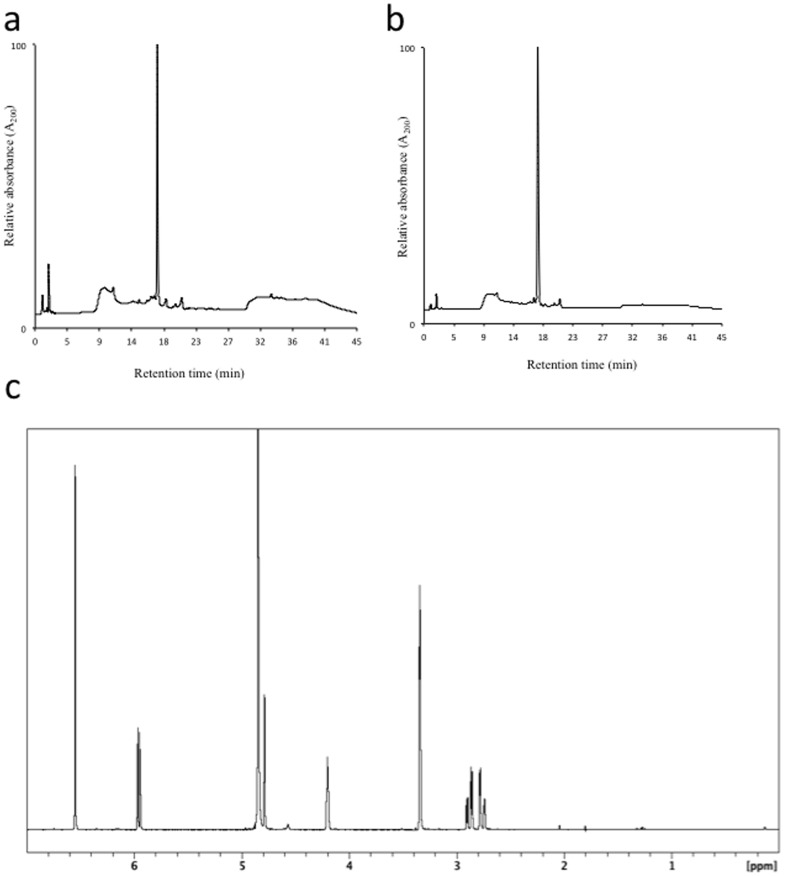
EGC reference standard from Nacalai USA (Product No. 0256454). (**a**) HPLC chromatogram of EGC from Nacalai USA; (**b**) HPLC chromatogram of a combined sample of EGC from Nacalai USA and EGC from Sigma-Aldrich Corp.; and (**c**) ^1^H-NMR spectrum, record in CD_3_OD at 400 MHz of EGC (ca. 5 mg/mL) from Nacalai USA (Product No. 0256454).

### HPLC Analysis

A Shimadzu (Columbia, MD, USA) Prominence HPLC system consisting of a photodiode array (PDA) detector and LC Solution software was used for all HPLC analyses. A 150×4.6 mm, 5 µm Ultra II reverse-phase C-18 column (Restek, Bellefonte, PA, USA), and a gradient elution of acetonitrile and ultrapure water were used for all HPLC separations. For all analyses the injection volume was 10 µL, with a flow rate of 1 mL/min and controlled oven temperature of 25°C.

For the quantitative HPLC analysis, individual calibration curves were created for each of the six standards; *L*-theanine, caffeine, EGCG, EGC, EC and ECG dissolved in a mixture of methanol and water (70∶30 v/v).

### Analytical Methods (NMR, MS, IR and UV/Vis Spectroscopy)

^1^H-NMR spectroscopy was performed with a Bruker (Billerica, MA, USA) Avance 400 MHz NMR spectrometer. Electrospray ionization mass spectrometry (ESI-MS) was performed on a Varian (Agilent Technologies, Santa Clara, CA, USA) 500-MS IT mass spectrometer. Fourier transform infra red (FT-IR) spectroscopy was done on a Thermo Scientific (Waltham, MA, USA) NICOLET iS10 IR equipped with a SMART iTR sampling accessory. UV/Vis spectroscopy data were taken from the HPLC PDA detector (see above: HPLC Analysis).

### Ferric Reducing Antioxidant Power (FRAP) Assay

The FRAP assay employed was modified from the Benzie and Strain protocol [4], and detailed in a previous publication [5].

## Results and Discussion

To confirm the identity and concentrations of *L*-theanine, caffeine, and the four catechin-polyphenols; EGCG, EGC (Fig. 1, Structure **1**), EC and ECG within the chromatograms obtained from the HPLC analysis of our tea leaf extracts it was necessary to use validated standards as references, in this case ones that were commercially available. In the course of the investigation it was found that one of these standards (EGC), although having the physical appearance and appropriate HPLC chromatographic behavior of EGC (i.e., HPLC retention time), was in actual fact a completely different class of molecule, *trans*-epoxysuccinyl-*L*-leucylamido-(4-guanidino)-butane (E-64), Fig. 1, Structure **2**. It should be noted that no stereochemical assignments are shown for **2** as some of our NMR data is at variance with assignments provided for this material.

The initial reference standard of EGC (≥95%, Product No. E3768) used was obtained from Sigma-Aldrich Corp., USA. The HPLC chromatogram of this reference standard is shown in Fig. 2a. This material had a retention time in accord with comparable HPLC chromatographic data reported in the literature for EGC [6]. Using this reference standard, as well as others obtained for this study, we were able to successfully assign appropriate peaks in the HPLC chromatograms acquired while analyzing various green tea extracts (e.g., Fig. 2b). With this part of the analysis complete we undertook the second part of the study that was aimed at correlating the observed changes in catechin-polyphenol concentrations between tea samples and their antioxidant activity as determined employing the FRAP assay [4]. When the FRAP activity of this EGC standard was tested it was found to be devoid of any antioxidant activity. This result was totally unexpected as EGC is well-known for its potent antioxidant activity [7], [8]. To ensure this result was not a false negative the FRAP assay was repeated twice more with this reference sample of EGC and a variety of known FRAP positive antioxidants. In each case, this EGC reference material showed no FRAP activity while the other compounds used were active as expected (data not shown). At this point it was decided to thoroughly investigate the reference standard sample by undertaking a full spectroscopic analysis. First, we performed what we consider to be the simplest, quickest, non-destructive, completely diagnostic spectroscopic measurement we could; recording of a ^1^H-NMR spectrum (Fig. 2c). This analysis was repeated several times, without HOD suppression at ambient temperature (ca. 22°C), with HOD suppression at ambient temperature (ca. 22°C), and then both measurements again at 25°C.

With these measurements complete (Fig. 2c) it was evident from the resultant spectrum that the sample we were assuming was EGC was a class of molecule in no way related to EGC, and was identified as *trans*-epoxysuccinyl-*L*-leucylamido-(4-guanidino)-butane (E-64) (Fig. 1, Structure **2**) even though compared to EGC it had a similar physical appearance and essentially identical HPLC behavior in the system we employed for our analyses. To further substantiate our deduction concerning the now supposed EGC reference we recorded mass (MW  = 357 amu compared to EGC  = 306 amu), IR (Fig. S1a) and UV-Vis [broad ever increasing signal with no specific λ_max_ 204 nm compared to EGC from green tea extract (Fig. 2b, peak 2) and an authentic reference λ_max_ 204 nm] spectra of the material. These data, especially the MS and IR (Fig. S1a), further confirmed that the reference sample from Sigma-Aldrich Corp., was not EGC, in contrast to what the Certificate of Analysis claimed (see Fig. S2). To further confirm the deductions concerning the reference standard we compared our NMR data to that reported in the literature for EGC [9]. The outcome of this comparison further confirmed our deduction. Now, convinced that the purchased and supposedly validated reference standard was indeed not EGC we considered the implications for all of our other standards and all of the qualitative and quantitative research we had undertaken over the years where standards were not absolutely validated. It also raised significant questions concerning the validity of many published pieces of research that used quantitative HPLC methods without first performing rigorous validation of the true nature of employed standards prior to their use; something we are now undertaking on a routine basis.

Following this finding we obtained a new reference standard of EGC (98%, Product No. 0256454) from Nacalai, USA. The HPLC chromatogram of the new reference standard is shown in Fig. 3a. This new reference standard had the typical HPLC behavior of EGC and was comparable to the appropriate HPLC chromatographic behavior of EGC reported in the literature [6]. HPLC performed on a sample that contained both the original reference standard (Sigma-Aldrich Corp., USA) and the new reference standard of EGC (Nacalai, USA) at the same concentrations showed the compounds to co-elute (Fig. 3b). Structure validation of the new reference standard of EGC using ^1^H-NMR (Fig. 3c) gave the expected spectrum of EGC compared to the data reported in the literature [9], as did its MS (MW  = 306 amu), IR (Fig. S1b) and UV-Vis data (λ_max_ 204 nm). Finally, antioxidant activity testing performed, using the FRAP assay, with the new reference standard of EGC resulted in an antioxidant activity of 236.7±0.7 µM FRAP value per µg EGC, which is comparable to its reported antioxidant activity [7], [8]. These experimental data proved we now had an authentic sample of EGC that could be used as a valid reference sample. This meant we could now repeat with confidence all of our original analyses made with the tea extracts.

This relatively simple example graphically illustrates the importance of performing a validation of all reference standards used in qualitative and quantitative analysis of all organic substances irrespective of their origin by recording at least a ^1^H-NMR spectrum of them prior to their use or by having the supplier provide this data for the supplied material.

Why a ^1^H-NMR spectrum and not some other measurement? The answer to this question relates purely and simply to the ease with which the measurement can be made, the high diagnostic and predictive content of measured data and the fact that no material is lost or destroyed in the measurement. All other measurements; HPLC, MS, UV, IR, etc., lack some degree of certainty concerning structure that ^1^H-NMR does not. Clearly, there will be exceptions to this generalization concerning ^1^H-NMR but compared to the other methods mentioned they will be insignificant by comparison.

In summary, from our fortunate experience of making more than one type of unrelated measurement with a reference material we were saved from reporting incorrect and misleading analytical data into the scientific literature. This experience has led us to review many of our laboratory practices and has convinced us that every new organic material that we obtain for use in our research will have at least a ^1^H-NMR spectrum record and this data will then be compared to known data for the same material. We would also encourage all other researchers, particularly those in the areas of qualitative and quantitative analysis and validation to review their processes and procedures and perhaps consider recording, or have recorded, routine ^1^H NMR spectra of all of their reference standards.

## Supporting Information

Figure S1 **Fourier transform infra red (FTIR) spectrum of (a) supposed EGC from Sigma-Aldrich, USA, and of (b) actual EGC from Nacalai, USA.**
(TIF)

Figure S2 **Certificate of Analysis of supposed EGC (Product No. E3768) from Sigma-Aldrich website (** http://www.sigmaaldrich.com/ **, Accessed 2011 Sep 20, 2011).**
(TIF)
