# Supplementary figures and images for: The Importance of 1H-Nuclear Magnetic Resonance Spectroscopy for Reference Standard Validation in Analytical Sciences
Source: PLoS One. 2012 Jul 27;7(7):e42061. doi: 10.1371/journal.pone.0042061 (PMC3407044; doi:10.1371/journal.pone.0042061)

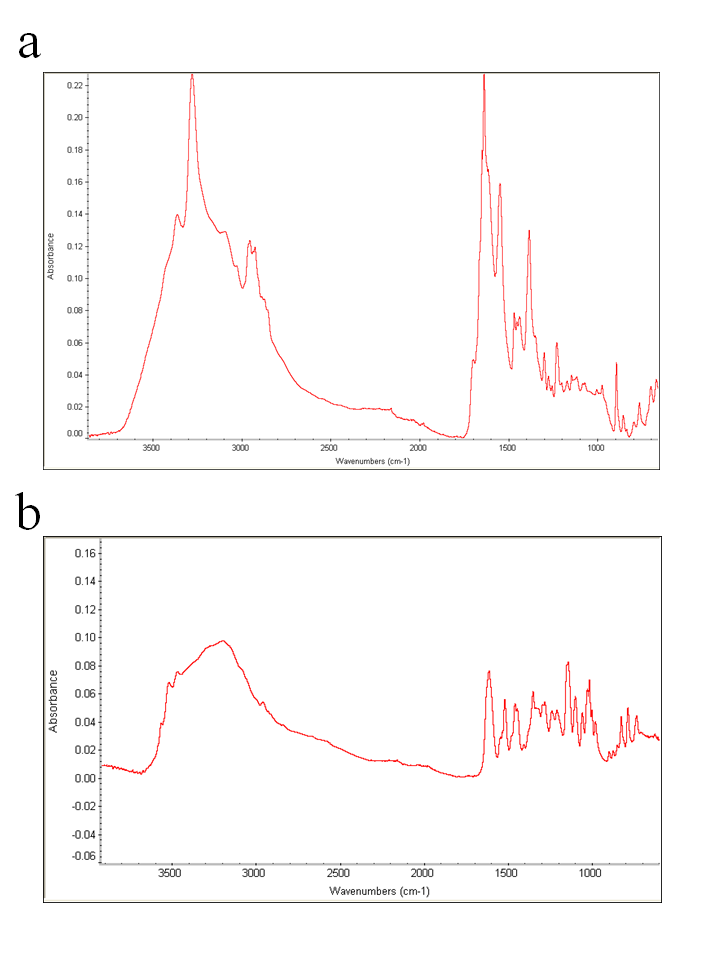

Supplement: Figure S1 — Fourier transform infra red (FTIR) spectrum of (a) supposed EGC from Sigma-Aldrich, USA, and of (b) actual EGC from Nacalai, USA. (TIF) [file pone.0042061.s001.tif]

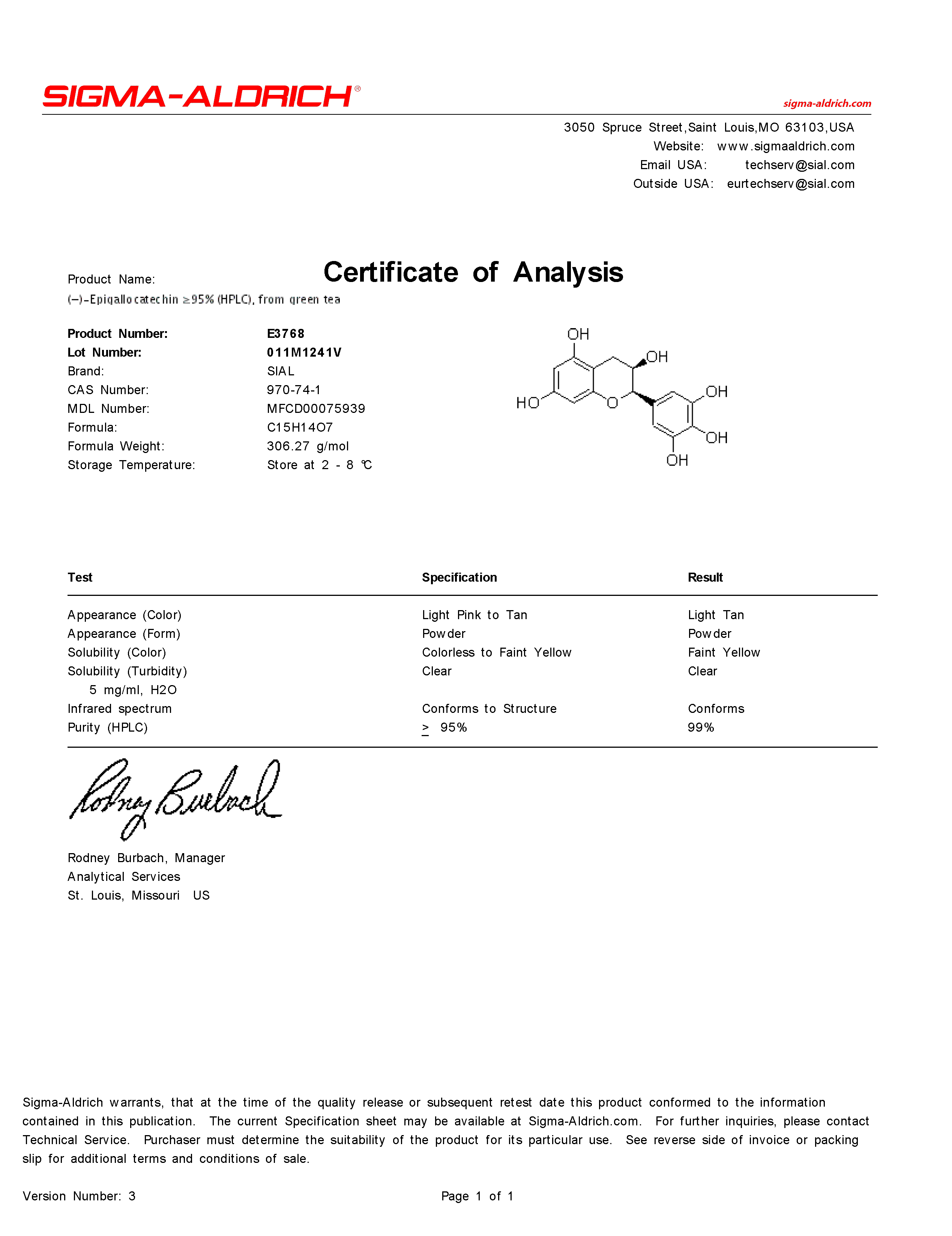

Supplement: Figure S2 — Certificate of Analysis of supposed EGC (Product No. E3768) from Sigma-Aldrich website ( http://www.sigmaaldrich.com/ , Accessed 2011 Sep 20, 2011). (TIF) [file pone.0042061.s002.tif]
